# Uncovering the Specific Functions of miR-33 in Regulation of Feeding and Cardiometabolic Diseases Linked to Aging

**DOI:** 10.1093/geroni/igaa057.421

**Published:** 2020-12-16

**Authors:** Nathan Price, Xinbo Zhang, Pablo Fernandez-Tussy, Rafael de Cabo, Carlos Fernandez-Hernando

**Affiliations:** 1 Yale University, New Haven, Connecticut, United States; 2 National Institute on Aging, Bethesda, Maryland, United States

## Abstract

Heart disease and metabolic dysfunction are two of the most important age related health issues, and feeding behavior is a critical factor contributing to these conditions. miR-33 promotes the development of atherosclerosis, by impairing macrophage cholesterol efflux and reverse cholesterol transport. Specific disruption of the interaction between miR-33 and the cholesterol transporter ABCA1 protected mice from atherosclerosis in a manner similar to that observed with loss or inhibition of miR-33. However, miR-33 has also been shown to impact other cellular functions, including targeting numerous mRNAs related to bioenergetics and inflammatory response, that may also contribute to its effects on atherosclerosis. Moreover, characterization of miR-33 deficient animals has revealed a strong predisposition to the development of obesity and metabolic dysfunction. While this phenotype appears to be due to alterations in feeding behavior, it is not clear what organ or organs are primarily driving this effect or what functions of miR-33 may be responsible. To address these questions, we have generated conditional miR-33 knockout mice to selectively remove miR-33 from a number of key metabolic tissues. Using these unique mouse models, we have performed an extensive characterization of how miR-33 impacts the function of different metabolic tissues in both chow and high fat diet fed mice, and assessed what impact this has on regulation of metabolic homeostasis and atherosclerosis. This work will improve our understanding of the mechanisms regulating feeding behavior and provide critical information for the development and evaluation of novel approaches to combat cardiometabolic diseases associated with aging.

